# Supplementary material for: Evolution of Omicron lineage towards increased fitness in the upper respiratory tract in the absence of severe lung pathology
Source: Nat Commun. 2025 Jan 11;16:594. doi: 10.1038/s41467-025-55938-3 (PMC11724920; doi:10.1038/s41467-025-55938-3)
Supplement: Supplementary file 1 — Supplementary Information [file 41467_2025_55938_MOESM1_ESM.pdf]

Supplementary Materials for:

Evolution of Omicron lineage towards increased fitness in the upper respiratory tract in the absence of severe lung pathology

Arthur Wickenhagen[1#], Meaghan Flagg[1#], Julia R Port[1,2], Claude Kwe Yinda[1], Kerry Goldin[1], Shane Gallogly[1], Jonathan E Schulz[1], Tessa Lutterman[1], Brandi N Williamson[1], Franziska Kaiser[1], Reshma K Mukesh[1], Sarah van Tol[1], Brian Smith[3], Neeltje van Doremalen[1], Colin A Russell[4], Emmie de Wit[1&], Vincent J Munster[1&]

This file includes:

Supplementary table 1. Summary of historical D614G, B.1.17, and Delta hamster infection studies
Supplementary table 2. Virus neutralization titers related to Figure 7a
Supplementary table 3. ISG primer/probe sequences
Supplementary figure 1. Cell line and pseudovirus generation.
Supplementary figure 2. Hamster weights and histological changes in hamster lungs after I.N. inoculation with different Omicron variants.
Supplementary figure 3. Cytotoxicity and epithelial barrier integrity following Omicron variant infection in nasal ALI cultures.
Supplementary figure 4. Hamster weights and histological changes in hamster lungs after I.T. inoculation with different Omicron variants.
Supplementary figure 5. Cytotoxicity of Omicron variants in human iPSC-derived alveolar organoids (ihLOs).
Supplementary figure 6. Pro-inflammatory cytokine gene expression in nasal ALI cultures.

Supplementary table 1. Summary of historical D614G, B.1.17, and Delta hamster intranasal infection studies

		Viral load (sgRNA mean log_{10} copies/mL or log_{10} copies/g)		Virus titer (mean log_{10} TCID50/mL or log_{10} $TCID_{50}$/g)	
		D3	D5	D3	D5
D614G[1]	Swab	N.D.	N.D.	4.22	2.47
	Lung	N.D.	N.D.	N.D.	6.80
B.1.1.7 (alpha)[2]	Swab	N.D.	N.D.	N.D.	N.D.
	Lung	N.D.	10.35	N.D.	6.88
Delta[3]	Swab	6.47	5.96	5.31	3.08
	Lung	9.83	9.75	9.77	7.79
Omicron XBB.1.5[4]	Swab	6.43	5.27	N.D.	N.D.
	Lung	N.D.	7.31	N.D.	2.37
Omicron XBB.1.16[4]	Swab	5.54	4.73	N.D.	N.D.
	Lung	N.D.	7.05	N.D.	2.57
Omicron EG.5.1[4]	Swab	5.75	5.18	N.D.	N.D.
	Lung	N.D.	7.58	N.D.	4.54
Omicron JN.1[4]	Swab	5.00	3.06	N.D.	N.D.
	Lung	N.D.	5.03	N.D.	0.50

[1]D614G: DOI:10.1126/scitranslmed.abh0755 (ref 22)
[2]B.1.1.7: DOI:10.1038/s41467-021-26178-y (ref 23)
[3]Delta: DOI:10.1038/s41467-022-32248-6 (ref 24)
[4]This study
N.D. data not available

Supplementary table 2. Virus neutralization titers related to Figure 7a

		Virus used in assay						
Animal #	Infected with	**WA-1**	**Alpha**	**BA.1**	**EG.5.1**	**XBB.1.5**	**XBB.1.16**	**JN.1**
1	XBB.1.16	20	<10	20	640	240	2560	40
2	XBB.1.16	30	<10	40	320	120	1280	40
3	XBB.1.16	10	<10	<10	160	80	1280	10
4	XBB.1.16	20	10	80	640	240	1280	60
5	XBB.1.16	<10	<10	30	240	160	640	40
6	XBB.1.16	<10	<10	20	240	240	2560	40
7	XBB.1.5	60	20	160	2560	960	960	120
8	XBB.1.5	10	20	240	640	320	640	160
9	XBB.1.5	60	20	320	1920	1280	640	240
10	XBB.1.5	20	<10	120	480	640	640	40
11	XBB.1.5	30	<10	30	960	320	320	60
12	XBB.1.5	20	<10	160	960	960	640	120
13	EG.5.1	<10	<10	<10	480	800	120	<10
14	EG.5.1	10	<10	60	480	800	240	<10
15	EG.5.1	<10	<10	40	1280	1200	320	10
16	EG.5.1	<10	<10	<10	320	600	160	<10
17	EG.5.1	<10	<10	<10	240	600	120	<10
18	EG.5.1	<10	<10	<10	240	400	80	<10
19	JN.1	<10	<10	<10	40	200	<10	480
20	JN.1	<10	<10	<10	<10	<10	<10	240
21	JN.1	<10	<10	<10	<10	<10	<10	240
22	JN.1	<10	<10	<10	10	<10	<10	320
23	JN.1	<10	<10	<10	<10	<10	<10	60
24	JN.1	<10	<10	<10	<10	<10	<10	60

Supplementary table 3. ISG primer/probe sequences.

Gene	Forward	Reverse	Probe
ISG15	GCCTTCAGCTCTGACACC	CGAACTCATCTTTGCCAGTACA	CACCTGGAATTCGTTGCCCGC
ISG20	CTCGCATCTTCCACCGA	AATCTACGACACGTCCACTG	TCACTCAGCACCCGCAGGG
MX1	AGCGCATCTCCAGCCACATC	GTGTCGCTCCGCTCCTTCAG	AGGCCATGCTGCAGCTCCTGCAGGA
OAS1	GTGCTGCCTGCCTTTGATGC	GGCGCTGCTTCAGGAAGTCT	TCGCCCTCTTTCTGCAGGTCGGTGCA
RIG1	CCGGAAGACCCTGGACCCTA	ACGGAACCAGCCTTCCTCCT	AGGGCCCAATGGAGGCTGCCACACT
IFIT1	GGGCAACTTTGCCTGGATGT	GCAAGGCCCATCCTTCCTCA	ACCACATGGGCAGACTGGCAGAAGCCC
IFITM3	AAAGCGTGTGAGGATAAAGGG	AGGCCTATGGATAGATCAGGAG	CGAGGAATGGAAGTTGGAGTACGTGG
IFNB	CAGCAGTTCCAGAAGGAGGA	AGCCAGGAGGTTCTCAACAA	CGCCGCATTGACCATCTATGAGATGC
IL6	ATGCCAGCCTGCTGACGAAG	AAGAGCCCTCAGGCTGGACT	CCTGCAGCCACTGGTTCTGTGCCTGC
IL1B	TGCACGATGCACCTGTACGA	TGTCCCTGGAGGTGGAGAGC	ACTGCACGCTCCGGGACTCACAGCA
TNFA	TCTGGCCCAGGCAGTCAGAT	ATTGGCCAGGAGGGCATTGG	CCCGGCGGTTCAGCCACTGGAGC

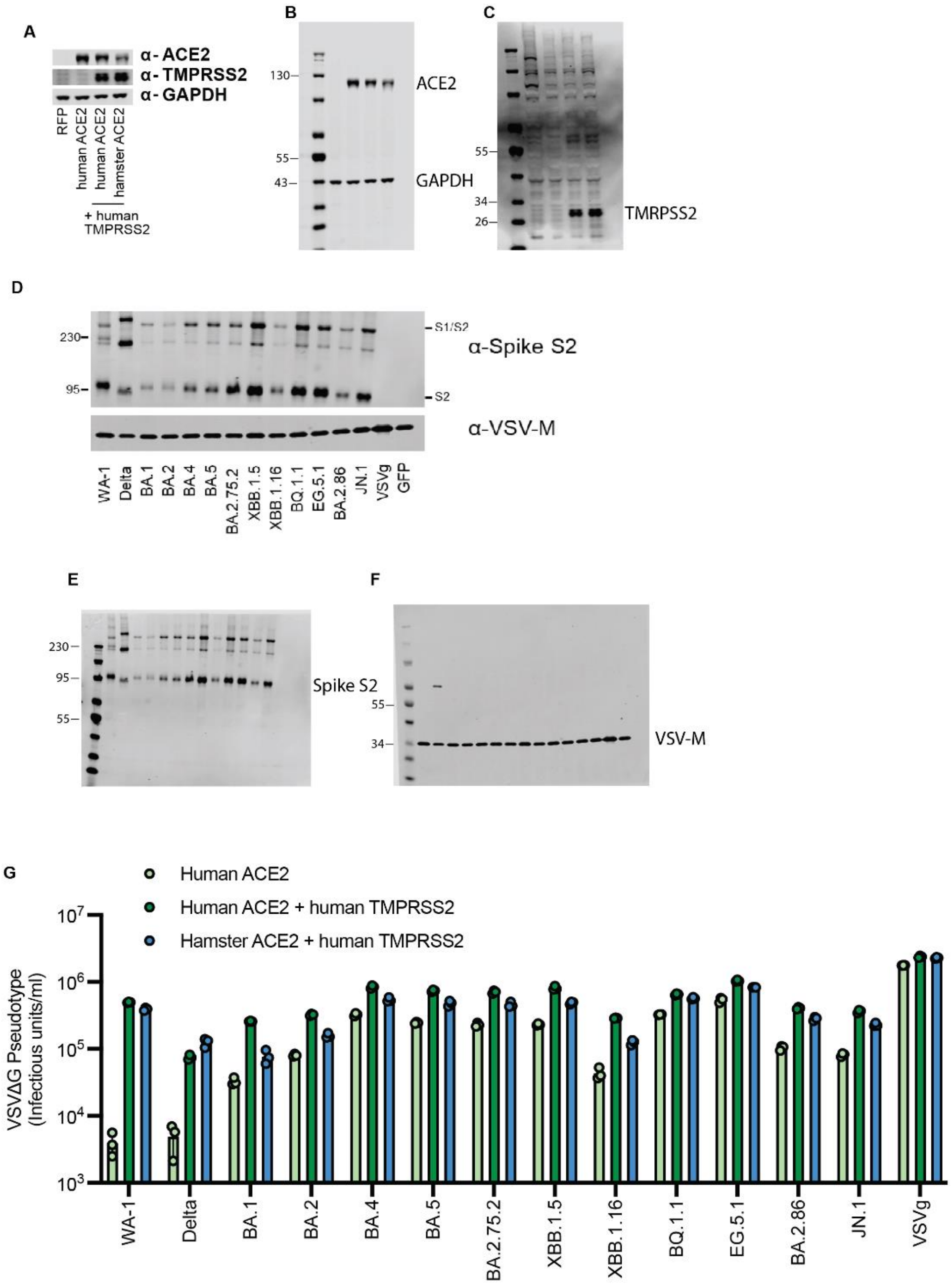

Figure S1. Cell line and pseudovirus generation. (A) A549 cells with stable expression of RFP control, human ACE2, human ACE2 and TMPRSS2 or hamster ACE2 and human TMPRSS2. Cell lysates were probed with polyclonal antibody against ACE2, TMPRSS2 or GAPDH. One blot of two independent experiments is shown. (**B-C**) Complete blots corresponding to S1A. **(D)** Pseudovirus supernatants were concentrated over a sucrose cushion and lysed in protein sample buffer. Lysates were probed with antibody against SARS-CoV-2 Spike S2 subunit or VSV-matrix protein. One blot of two independent experiments is shown. (**E-F**) Complete blots corresponding to S1D. (**G**) Pseudovirus entry titers (infectious units/mL) underlying fold change calculations shown in Figure 1, represented as mean +/- S.D. of n=3 biological replicates.

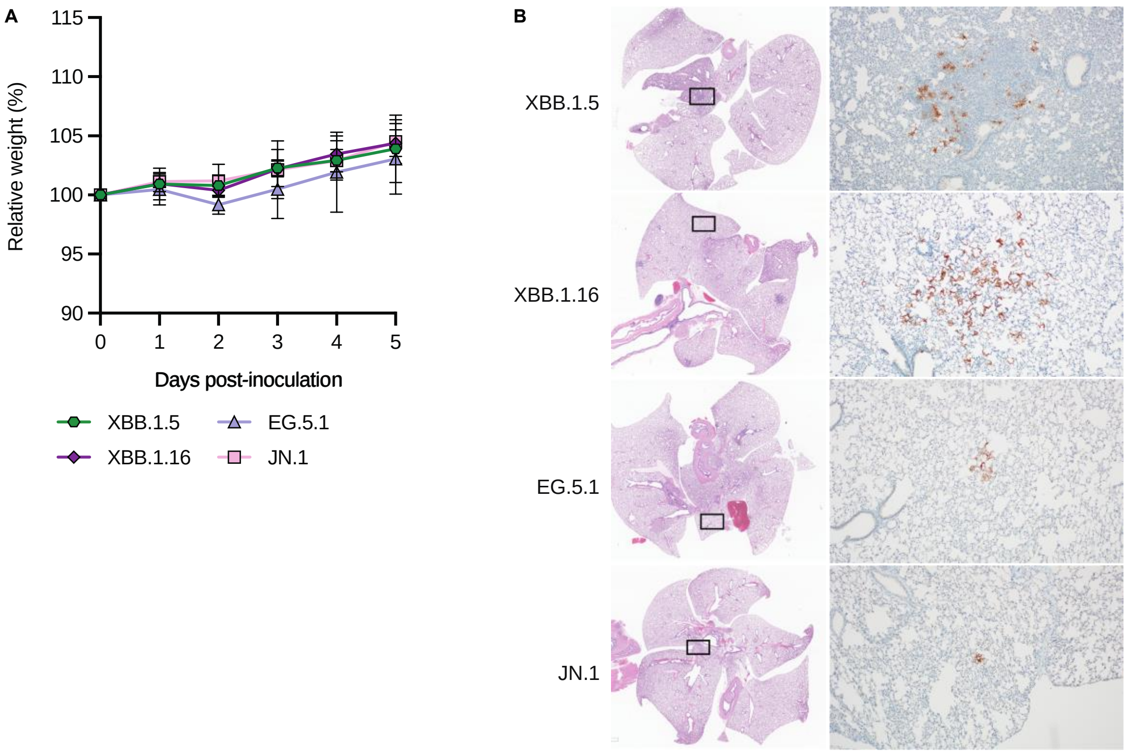

Figure S2. Hamster weights and histological changes in hamster lungs after I.N. inoculation with different Omicron variants. (A) Relative weight of hamsters challenged intranasally (I.N.) with 10^4 $TCID_{50}$ in comparison to day 0. Error bars denote mean +/- SD of n=4 animals. **(B)** Hematoxylin and eosin stain (H&E) and immunohistochemistry (IHC) for SARS-CoV-2 NP was performed on lungs of hamsters inoculated I.N. with indicated Omicron variants at 5DPI. H&E (left column) scale bars = 3mm, black square on H&E indicates field of IHC staining. IHC (right column) scale bars = 200 µm.

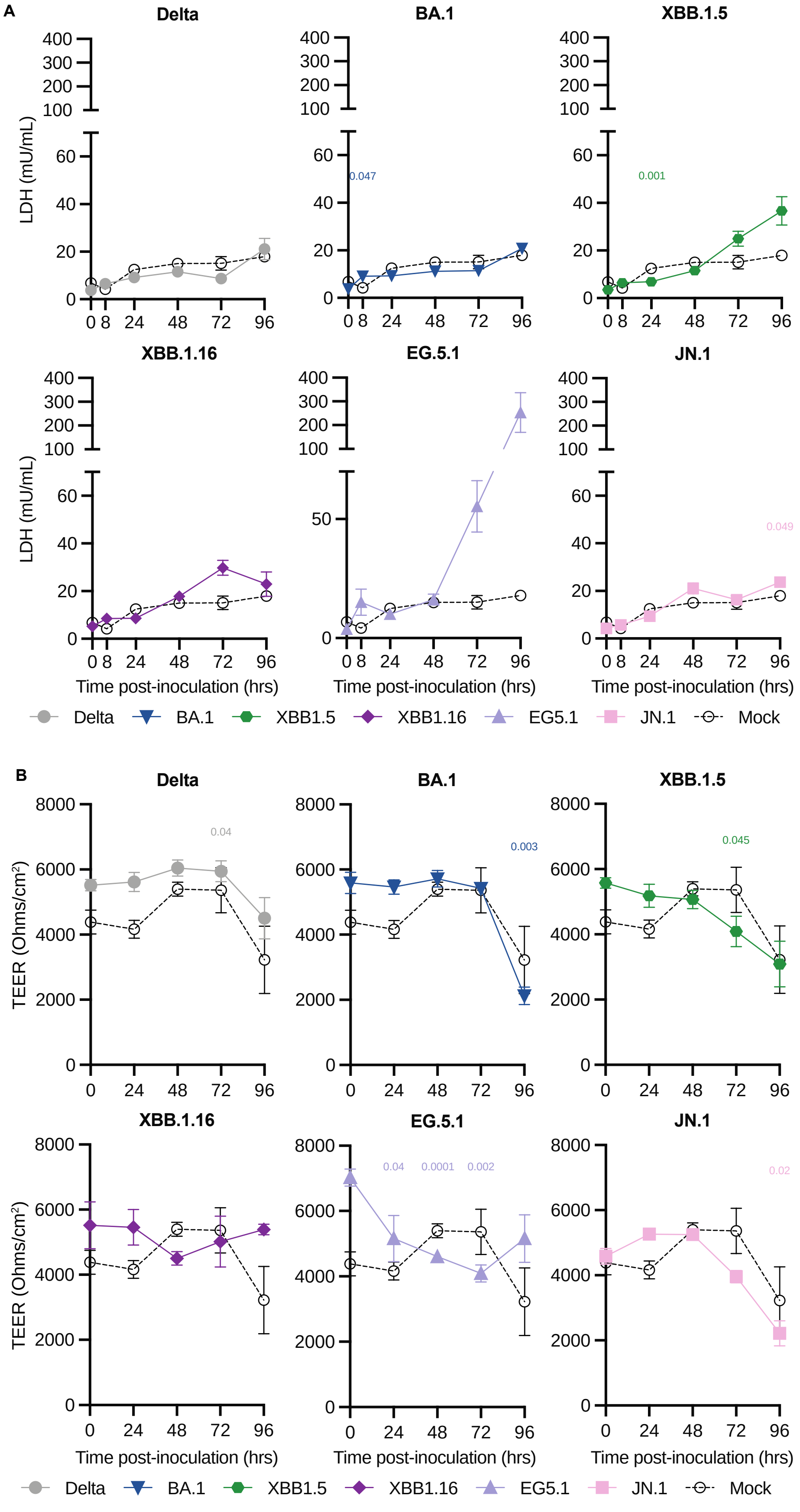

A
Delta
BA.1
XBB.1.5
XBB.1.16
EG.5.1
JN.1
LDH (mU/mL)
0.047
0.001
0.049
Time post-inoculation (hrs)
Delta
BA.1
XBB1.5
XBB1.16
EG5.1
JN.1
Mock
B
Delta
BA.1
XBB.1.5
XBB.1.16
EG.5.1
JN.1
TEER (Ohms/cm²)
0.04
0.003
0.045
0.04
0.0001
0.002
0.02
Time post-inoculation (hrs)
Delta
BA.1
XBB1.5
XBB1.16
EG5.1
JN.1
Mock

Figure S3. Cytotoxicity and epithelial barrier integrity following Omicron variant infection in nasal ALI cultures. (A) Cytotoxicity was measured via LDH release into culture supernatant. Error bars denote mean +/- SEM of n=7 (0-48h) or n=4 (72-96h) biological replicates. **(B)** TEER was measured over the course of infection. P-values denote difference vs 0 hours post-infection. Statistical analysis was conducted using restricted maximum likelihood mixed effects model followed by Dunnett's post-test. P-values < 0.05 are shown.

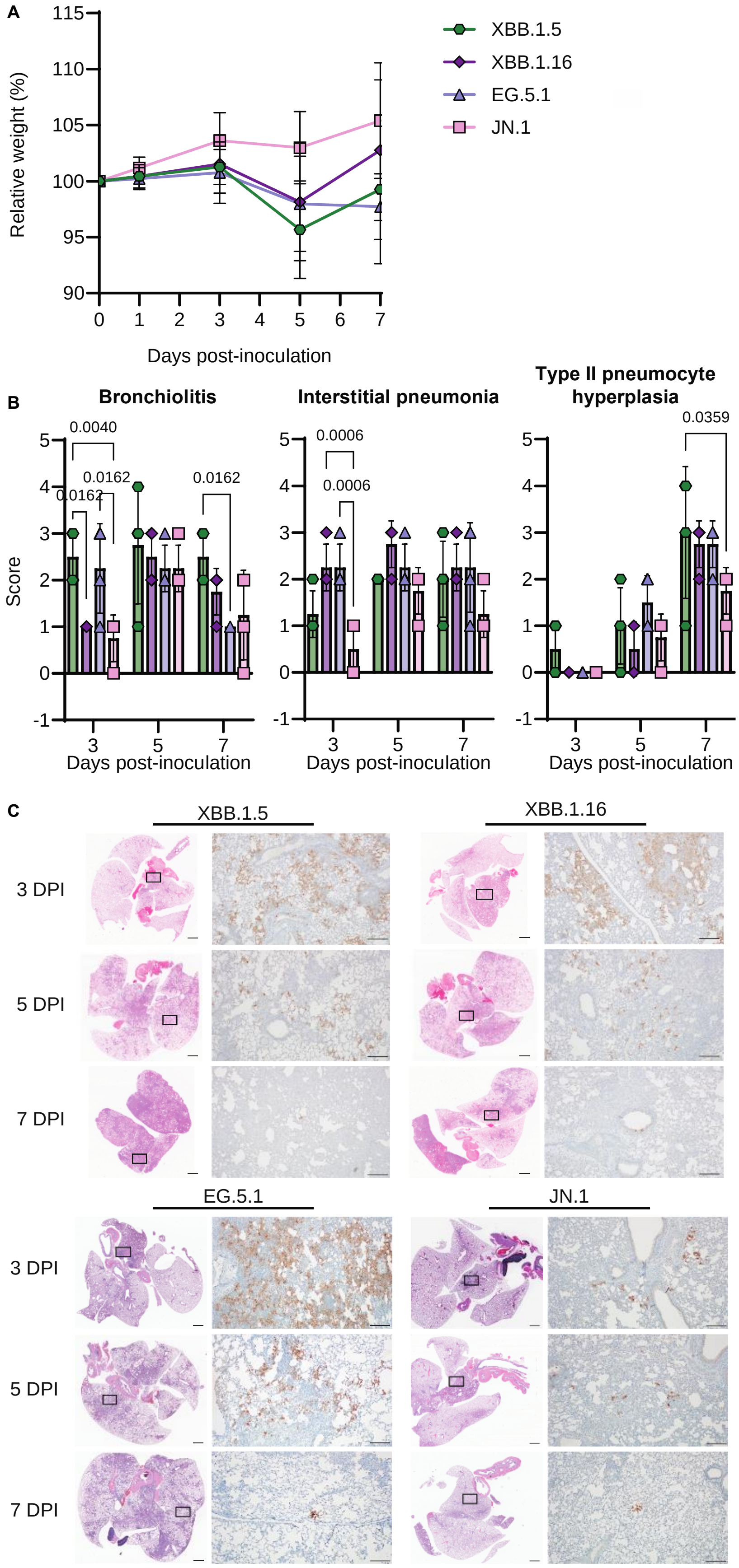
A
B
C
Relative weight (%)
Days post-inoculation
XBB.1.5
XBB.1.16
EG.5.1
JN.1
Bronchiolitis
Interstitial pneumonia
Type II pneumocyte hyperplasia
Score
0.0040
0.0162
0.0162
0.0162
0.0006
0.0006
0.0359
3 DPI
5 DPI
7 DPI

Figure S4. Hamster weights and histological changes in hamster lungs after I.T. inoculation with different Omicron variants. (A) Relative weight of hamsters challenged intratracheally (I.T.) with 10^4 $TCID_{50}$ in comparison to day 0. Error bars denote mean +/- SD of n=6 animals, except EG.5.1 n=4. **(B)** Semi-quantitative scores were assigned to histopathological changes in the lungs of hamsters by a board-certified veterinary anatomic pathologist. Error bars denote mean +/- SD. Statistical analysis was conducted using two-way ANOVA followed by Tukey's post-test and P-values < 0.05 are shown. **(C)** Slides for hematoxylin and eosin stain (H&E) and immunohistochemistry (IHC) for SARS-CoV-2 from hamsters inoculated I.T. with indicated variants at 3, 5, and 7 DPI. H&E (left column) scale bars = 3mm, black square on H&E indicates field of IHC staining. IHC (right column) scale bars = 200 μm.

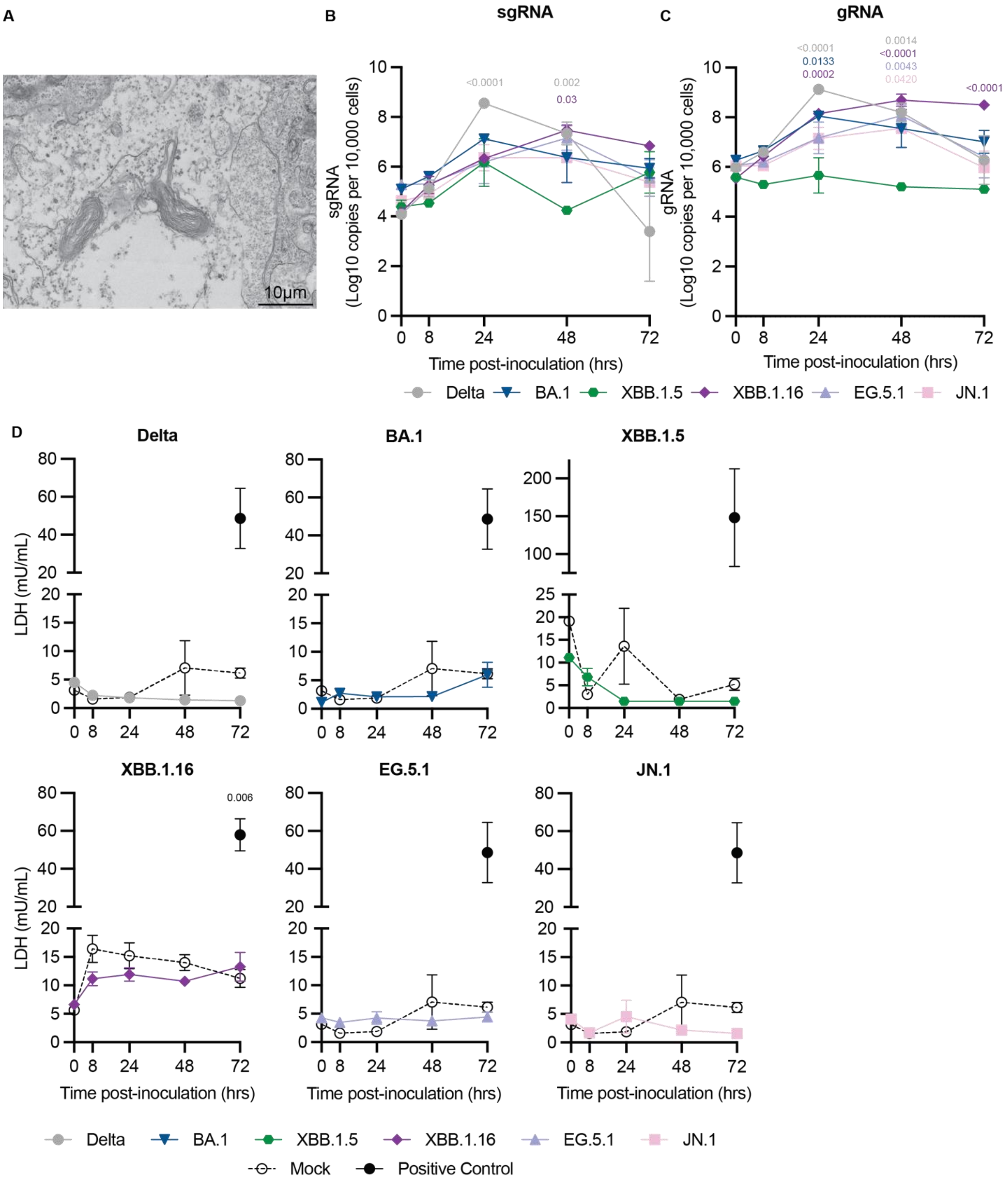

Figure S5. Cytotoxicity of Omicron variants in human iPSC-derived alveolar organoids (ihLOs). (A) Transmission electron micrograph demonstrating presence of lamellar bodies in ihLOs. **(B-C)** Viral load in cell lysate was quantified by qRT-PCR for SARS-CoV-2 sgRNA (B) or gRNA (C). A dilution series of RNA standards with known copy numbers was run in parallel to calculate copy numbers in the samples. P-values denote difference vs 0 hours post-infection. **(D)** Cytotoxicity was evaluated by measuring LDH release into culture supernatant. Lysis of organoids with 0.2% Triton X-100 was included as a positive control for maximal LDH release.

P-values denote difference vs mock-infected controls at the corresponding time point. Experiments involving XBB.1.5 and XBB.1.16 were run separately alongside matched mock-infected and positive controls. Error bars denote mean +/- SEM of n=3 biological replicates. Statistical analysis was conducted using two-way ANOVA followed by Dunnett's post-test (B-D), or with unpaired T test to compare positive controls to mock-infected samples at 72 hours post-infection (D). P-values < 0.05 are shown.

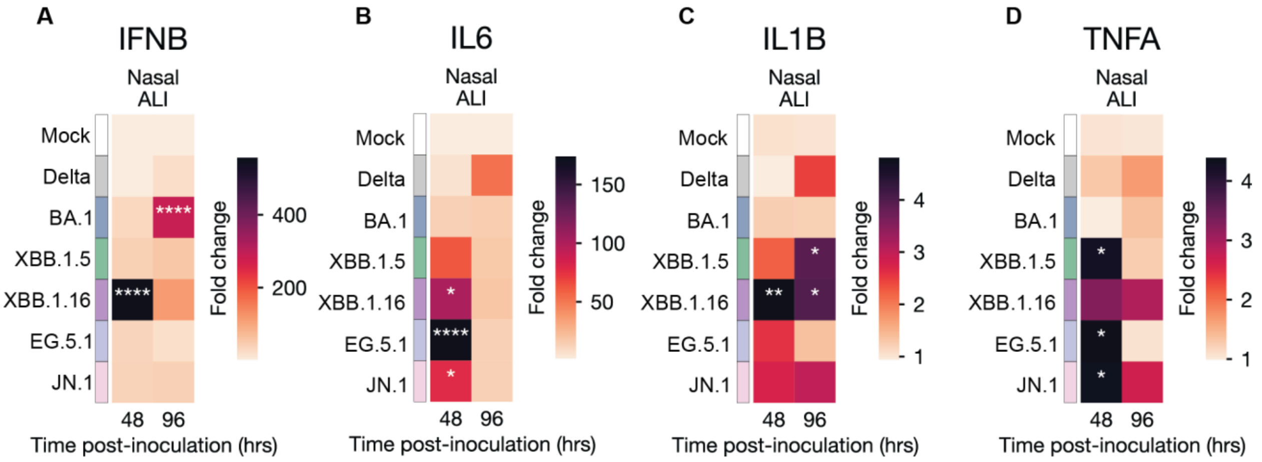

Figure S6. Pro-inflammatory cytokine gene expression in nasal ALI cultures. (A-D) Expression of pro-inflammatory cytokines in SARS-CoV-2-infected nasal ALI cultures was determined by qRT-PCR. Fold change in gene expression was calculated relative to timepoint-matched mock-infected controls. Mean fold change of n=3 biological replicates is shown. Statistical analysis was conducted using one-way ANOVA with Dunnett's post-test. P-values < 0.05 for comparisons versus mock-infected controls are indicated. **** < 0.0001, *** < 0.001, ** < 0.01, * <0.05.
