# Supplementary material for: Evolution of Omicron lineage towards increased fitness in the upper respiratory tract in the absence of severe lung pathology
Source: Nat Commun. 2025 Jan 11;16:594. doi: 10.1038/s41467-025-55938-3 (PMC11724920; doi:10.1038/s41467-025-55938-3)
Supplement: Supplementary file 3 — Reporting Summary [file 41467_2025_55938_MOESM3_ESM.pdf]

nature portfolio

Corresponding author(s): Dr. Emmie de Wit and Dr. Vincent Munster

Last updated by author(s): Nov 13, 2024

Reporting Summary

Nature Portfolio wishes to improve the reproducibility of the work that we publish. This form provides structure for consistency and transparency in reporting. For further information on Nature Portfolio policies, see our Editorial Policies and the Editorial Policy Checklist.

Statistics

For all statistical analyses, confirm that the following items are present in the figure legend, table legend, main text, or Methods section.

n/a	Confirmed	
☐	☒	The exact sample size (*n*) for each experimental group/condition, given as a discrete number and unit of measurement
☐	☒	A statement on whether measurements were taken from distinct samples or whether the same sample was measured repeatedly
☐	☒	The statistical test(s) used AND whether they are one- or two-sided<br>*Only common tests should be described solely by name; describe more complex techniques in the Methods section.*
☒	☐	A description of all covariates tested
☐	☒	A description of any assumptions or corrections, such as tests of normality and adjustment for multiple comparisons
☐	☒	A full description of the statistical parameters including central tendency (e.g. means) or other basic estimates (e.g. regression coefficient) AND variation (e.g. standard deviation) or associated estimates of uncertainty (e.g. confidence intervals)
☐	☒	For null hypothesis testing, the test statistic (e.g. *F*, *t*, *r*) with confidence intervals, effect sizes, degrees of freedom and *P* value noted<br>*Give P values as exact values whenever suitable.*
☒	☐	For Bayesian analysis, information on the choice of priors and Markov chain Monte Carlo settings
☒	☐	For hierarchical and complex designs, identification of the appropriate level for tests and full reporting of outcomes
☒	☐	Estimates of effect sizes (e.g. Cohen's *d*, Pearson's *r*), indicating how they were calculated

Our web collection on statistics for biologists contains articles on many of the points above.

Software and code

Policy information about availability of computer code

Data collection	No software was used for data collection.
Data analysis	GraphPad Prism software, version 9.5.1;<br>Antigenic maps were constructed using antigenic cartography software from https://acmacs-web.antigenic-cartography.org.

For manuscripts utilizing custom algorithms or software that are central to the research but not yet described in published literature, software must be made available to editors and reviewers. We strongly encourage code deposition in a community repository (e.g. GitHub). See the Nature Portfolio guidelines for submitting code & software for further information.

Data

Policy information about availability of data

All manuscripts must include a data availability statement. This statement should provide the following information, where applicable:

- Accession codes, unique identifiers, or web links for publicly available datasets
- A description of any restrictions on data availability
- For clinical datasets or third party data, please ensure that the statement adheres to our policy

Data included in this manuscript have been deposited in Figshare at https://doi.org/10.6084/m9.figshare.26035741

Research involving human participants, their data, or biological material

Policy information about studies with human participants or human data. See also policy information about sex, gender (identity/presentation), and sexual orientation and race, ethnicity and racism.

Reporting on sex and gender	N/A
Reporting on race, ethnicity, or other socially relevant groupings	N/A
Population characteristics	N/A
Recruitment	N/A
Ethics oversight	N/A

Note that full information on the approval of the study protocol must also be provided in the manuscript.

Field-specific reporting

Please select the one below that is the best fit for your research. If you are not sure, read the appropriate sections before making your selection.

☒ Life sciences ☐ Behavioural & social sciences ☐ Ecological, evolutionary & environmental sciences

For a reference copy of the document with all sections, see nature.com/documents/nr-reporting-summary-flat.pdf

Life sciences study design

All studies must disclose on these points even when the disclosure is negative.

Sample size	Animal study sample size was determined by power analysis based on preexisting data.<br>For in vitro experiments, n>=3 replicates were used to enable statistical testing. Exact sample sizes are provided in figure legends.
Data exclusions	No data was excluded from this study.
Replication	All animal experiments were performed one time. In vitro experiments are one representative of two independent experiments, except for nasal air liquid interface cultures which were performed once due to technical limitations.
Randomization	Animals were allocated at random.
Blinding	Group allocation was not blinded. The following analysis tasks were blinded: histopathology and immunohistochemistry analysis

Reporting for specific materials, systems and methods

We require information from authors about some types of materials, experimental systems and methods used in many studies. Here, indicate whether each material, system or method listed is relevant to your study. If you are not sure if a list item applies to your research, read the appropriate section before selecting a response.

Materials & experimental systems

n/a	Involved in the study	
☐	☒	Antibodies
☐	☒	Eukaryotic cell lines
☒	☐	Palaeontology and archaeology
☐	☒	Animals and other organisms
☒	☐	Clinical data
☒	☐	Dual use research of concern
☒	☐	Plants

Methods

n/a	Involved in the study	
☒	☐	ChIP-seq
☐	☒	Flow cytometry
☒	☐	MRI-based neuroimaging

Antibodies

Antibodies used	anti-NP-1 antibody (GenScript U864YFA140-4/CB2093 NP-1)<br>anti-rabbit IgG polymer (Vector Laboratories # MP-6401)<br>goat anti-hamster IgG antibody (seracare CAT# 5220-0371)<br>anti-ACE2 (21115-1-AP, Proteintech)

	anti-TMPRSS2 (ab109131, Abcam)<br>anti-GAPDH (60004-1-Ig, Proteintech)<br>anti-SARS-CoV-2 spike S2 (MAB10557, R&D systems)<br>anti-VSV-M[23H12] (Ab01404-2.0, Absolute Antibody)<br>goat anti-rabbit IgG (SA5-10036, Thermo Fisher Scientific)<br>goat anti-mouse IgG (35519 or SA5-10176, Thermo Fisher Scientific)<br>anti-c-kit-PE (clone 104D2, BioLegend)<br>anti-CXCR4-Brilliant Violet 605 (clone 12G5, BioLegend)<br>anti-CD47-Brilliant Violet 421 (clone CC2C6, BioLegend)<br>anti-CD26-APC (clone BA5b, BioLegend)<br>anti-NKX2.1 (clone EP1584Y, Abcam)<br>anti-CPM (clone WK, FUJIFILM Wako Chemicals)<br>anti-rabbit IgG-PE (12-4739-81, Thermo Fisher Scientific)<br>anti-mouse IgG-PE (12-4010-82 Thermo Fisher Scientific).
Validation	Each goat anti-hamster IgG antibody lot is tested to assure specificity and lot-to-lot consistency using an in-house ELISA assay.<br>Anti-NP-1 antibody validation of cross-reactivity for SARS-CoV and SARS-CoV-2 in IHC was done in-house by embedding SARS-CoV-2 infected Vero cells in a histogel and producing and staining histology slides.<br>Validation of anti-c-kit-PE, anti-CXCR4-Brilliant Violet 605, anti-CD47-Brilliant Violet 421, anti-CD26-APC, anti-NKX2.1, anti-CPM is described in A. Jacob et al., Derivation of self-renewing lung alveolar epithelial type II cells from human pluripotent stem cells. Nat Protoc 14, 3303-3332 (2019).<br>anti-ACE2, anti-TMPRSS2, anti-GAPDH, anti-SARS-CoV-2 spike S2, anti-VSV-M were validated by the manufacturer for WB.

Eukaryotic cell lines

Policy information about cell lines and Sex and Gender in Research

Cell line source(s)	A549 (CCL-185), HEK293T (CRL-3216) and VeroE6 (CRL-1586) were sourced from ATCC. VeroE6-TMPRSS2-T2A-ACE2 (NR-54970) were sourced from BEI resources. Human iPSCs were sourced from Gibco,#A18945.
Authentication	None of the cell lines were authenticated.
Mycoplasma contamination	All cell lines tested negative for Mycoplasma contamination.
Commonly misidentified lines (See ICLAC register)	No commonly misidentified cell lines were used.

Animals and other research organisms

Policy information about studies involving animals; ARRIVE guidelines recommended for reporting animal research, and Sex and Gender in Research

Laboratory animals	Syrian hamsters (Mesocricetus auratus) 4-6 weeks old, mixed sex, sourced from Envigo.
Wild animals	N/A
Reporting on sex	no sex-based analysis was performed.
Field-collected samples	N/A
Ethics oversight	Rocky Mountain Laboratories Institutional Animal Care and Use Committee, NIAID,NIH.

Note that full information on the approval of the study protocol must also be provided in the manuscript.

Plants

Seed stocks	N/A
Novel plant genotypes	N/A
Authentication	N/A

Flow Cytometry

Plots

Confirm that:

☐ The axis labels state the marker and fluorochrome used (e.g. CD4-FITC).

☐ The axis scales are clearly visible. Include numbers along axes only for bottom left plot of group (a 'group' is an analysis of identical markers).

☐ All plots are contour plots with outliers or pseudocolor plots.

☐ A numerical value for number of cells or percentage (with statistics) is provided.

Methodology

Sample preparation	Cell culture fixed in PFA
Instrument	BD FACS Symphony
Software	BD FACS Diva<br>FlowJo v10.8.1
Cell population abundance	N/A
Gating strategy	FSC/SSC, SSC-w/SSC-h, FSC/GFP

☐ Tick this box to confirm that a figure exemplifying the gating strategy is provided in the Supplementary Information.
